# Knowledge and understanding of obstetric danger signs among pregnant women attending the antenatal clinic at the National Referral Hospital in Thimphu, Bhutan: a cross-sectional study

**DOI:** 10.1186/s12884-021-03580-4

**Published:** 2021-02-02

**Authors:** Saran Tenzin Tamang, Thinley Dorji, Sonam Yoezer, Thinley Phuntsho, Phurb Dorji

**Affiliations:** 1 Faculty of Postgraduate Medicine, Khesar Gyalpo University of Medical Sciences of Bhutan, Gongphel Lam, Thimphu, 11001 Bhutan; 2 Department of Internal Medicine, Armed Forces Medical College, Maharashtra University of Medical Sciences, Pune, India; 3 Kidu Mobile Medical Unit, His Majesty’s People’s Project, Thimphu, Bhutan; 4 Department of Obstetrics and Gynaecology, Khesar Gyalpo University of Medical Sciences of Bhutan, Thimphu, Bhutan

**Keywords:** Pregnancy, Prenatal care, Prenatal education, Maternal-child health services

## Abstract

**Background:**

The third Sustainable Development Goal for 2030 development agenda aims to reduce maternal and newborn deaths. Pregnant women’s understanding of danger signs is an important factor in seeking timely care during emergencies. We assessed knowledge of obstetric danger signs using both recall and understanding of appropriate action required during obstetric emergencies.

**Methods:**

This was a cross-sectional study among pregnant women attending antenatal clinic at Bhutan’s largest hospital in Thimphu. Recall was assessed against seven obstetric danger signs outlined in the Mother and Child Health Handbook (7 points). Understanding of danger signs was tested using 13 multiple choice questions (13 points). Knowledge was scored out of 20 points and reported as ‘good’ (≥80%), ‘satisfactory’ (60–79%) and ‘poor’ (< 60%). Correlation between participant characteristics and knowledge score as well as number of danger signs recalled was tested using Pearson’s correlation coefficient. Association between knowledge score and participant characteristics was tested using t-tests (and Kruskal-Wallis test) for numeric variables. Socio-demographic and clinical characteristics associated with the level of knowledge ('good’ versus ‘satisfactory’ and ‘poor’ combined) were assessed with odds ratios using a log-binomial regression model. All results with *p* < 0.05 were considered significant.

**Results:**

Four hundred and twenty-two women responded to the survey (response rate = 96.0%). Mean (±SD) knowledge score was 12 (±2.5). Twenty women (4.7%) had ‘good’ knowledge, 245 (58.1%) had ‘satisfactory’ knowledge and 157 (37.2%) had ‘poor’ knowledge. The median number of danger signs recalled was 2 (IQR 1, 3) while 68 women (20.3%) could not recall any danger signs. Most women were knowledgeable about pre-labour rupture of membranes (96.0%) while very few women were knowledgeable about spotting during pregnancy (19.9%). Both knowledge score and number of danger signs recalled had significant correlation with the period of gestation. Women with previous surgery on the reproductive tract had higher odds of having ‘good’ level of knowledge.

**Conclusions:**

Most pregnant women had ‘satisfactory’ knowledge score with poor explicit recall of danger signs. However, women recognized obstetric emergencies and identified the appropriate action warranted.

**Supplementary Information:**

The online version contains supplementary material available at 10.1186/s12884-021-03580-4.

## Background

The Sustainable Development Goals highlight the interconnectedness of maternal and neonatal health with other aspects of development [1]. The third SDG for 2030 aims to reduce preventable maternal deaths to < 70 per 100,000 live births and reduce neonatal mortality rates to 12 per 1000 live births [1]. Although most maternal deaths are preventable [2], it poses major challenges to health systems around the world, especially in low- and middle-income economies [3]. Global estimates of maternal mortality ratio (MMR) of 216 per 100,000 live births in 2015 and the neonatal mortality rate of 18 per 1,000 live births in 2017 reflect the need for concerted efforts in reducing preventable maternal and neonatal deaths [4, 5].

While many countries struggled to achieve the Millennium Development Goal related to reducing maternal mortality [3, 4], Bhutan was among the nine countries that achieved the greatest relative reduction in MMR [4]. MMR in Bhutan decreased from 560 in 1990 to 86 per 100,000 live births in 2012 [6]. This resulted from many policy and infrastructural reforms initiated by the Royal Government over the years. Key milestones include the Safe Motherhood and Child Survival Programme initiated in 1994, Emergency Obstetric Care Centres established in 1999, maternal mortality reviews started in 2000, Mother and Child Health (MCH) Handbook introduced in 2007, National Child Health Strategy developed in 2014, and the Bhutan Every Newborn Action Plan adopted in 2016 [7, 8]. In addition, health professionals are continually trained in delivering quality obstetric care and in prevention and management of emergencies such as post-partum haemorrhages [7, 8].

Over the years, the antenatal care uptake in Bhutan increased from 18.9% in 2000 to 97.9% in 2012 [7, 9]. By 2018, antenatal care uptake was > 95% with 31% ‘booking’ their pregnancy during the first trimester [10]. Skilled birth attendance and institutional delivery increased from 10.4% in 1994 to 73.7% in 2012 [7, 11]. However, given the relatively smaller birth cohort, maternal mortality has a huge bearing on the reporting of MMR targets. A review of maternal deaths between 2000 and 2017 in Bhutan reported that direct obstetric causes – post-partum haemorrhage, puerperal sepsis and pre-eclampsia – accounted for almost two-thirds of maternal deaths [7]. In 2021, with improved transportation network in the country, more than 95% of the population can access the health service within three hours of travel. However, additional efforts are required to further reduce preventable maternal and neonatal deaths to achieve the maternal mortality and neonatal mortality targets by 2030.

In order to reduce the consequences of ‘first delay’ in seeking care during obstetric emergencies, a pregnant woman’s knowledge and understanding of danger signs during pregnancy is vital in seeking timely care [2]. Studies in other settings have assessed knowledge by women’s ability to recall danger signs [12–15]. Although recall indicates that a woman has heard of danger signs, what action she would take if faced with emergency situations depends on her understanding of the danger signs. We assessed pregnant women’s knowledge of obstetric danger signs using both their ability to recall danger signs as well as their understanding of appropriate action necessary during potential obstetric emergencies.

## Methods

### Study design

This was a cross-sectional study among pregnant women attending antenatal clinic at Bhutan’s largest hospital, Jigme Dorji Wangchuck National Referral Hospital, between May 2019 and July 2020.

### Study setting

#### General setting

Bhutan is situated in the eastern Himalayas with a population of 0.7 million; Thimphu is its capital and largest city [16]. In 2017, Bhutan had 196,297 women in the reproductive age group (15–49 years) with 24,846 residing in Thimphu [16]. The national general fertility and total fertility rates were 57.3 and 1.7 per 1000 women, respectively [16].

The government provides free healthcare services across all levels – primary, secondary, and tertiary [10]. MCH services including antenatal care (ANC) and post-natal care are provided free of cost through hospitals, primary health centres and out-reach clinics [10]. As part of its Reproductive, Maternal, Neonatal and Child Health Programme, the Ministry of Health introduced the MCH Handbook in 2007, and revised it in 2014 and 2019 [10, 17].

The MCH Handbook is a recording tool as well as an information booklet. The handbook records parents’ demographic details, mother’s antenatal records, birth preparedness plan, birth details of the child, maternal and neonatal records, child’s growth charts, and vaccination records. It provides information on breastfeeding and nutrition, vaccination schedules, obstetric danger signs, monitoring developmental milestones, dental care, and general advice on antenatal and postnatal care [18].

A pregnant woman is issued the MCH Handbook during her ‘booking’ visit. Each Handbook has a unique identification number that tracks the pregnancy through the *Druk* Health Management and Information System [17]. Health workers explain the contents of the MCH Handbook during each antenatal visit. Staffs deliver key health messages in their daily talk (lasting 15–20 minutes) to the group of clients gathered in their respective units. Obstetric danger signs are discussed in these talks by health workers (health assistants, nurses or midwives). These health messages are reinforced during subsequent ANC and postnatal visits.

The MCH Handbook of Bhutan outlines seven key obstetric danger signs: 1) vaginal bleeding, 2) high fever, 3) preterm labour, 4) severe abdominal pain or vomiting, 5) severe headache, blurred vision or convulsions, 6) fast or difficult breathing, and 7) reduced or absent foetal movements.

#### Specific setting

This study was conducted at the Gyaltsuen Jetsun Pema Mother and Child Hospital, which is a part of the Jigme Dorji Wangchuck National Referral Hospital complex in Thimphu.

### Study participants

Pregnant women aged ≥18 years and attending ANC clinic at Gyaltsuen Jetsun Pema Mother and Child Hospital were eligible for the study. Participants were selected using systematic random sampling: every third pregnant woman registering for their routine ANC visit for the day was invited to participate in the study; those who consented were interviewed. Repetition of study participants was avoided by careful assessment of their MCH tracking number.

### Variables and data sources

Data were collected through interviewer-administered questionnaire that was designed for this study (Supplementary material). The questionnaire was pilot-tested among 20 pregnant women at Gyaltsuen Jetsun Pema Mother and Child Hospital in March 2019.

Pregnant women were asked to recall the danger signs outlined in the MCH Handbook (7 points). Their understanding of the danger signs was evaluated with 13 multiple choice questions – each question presented an obstetric emergency situation related to the danger signs outlined in the MCH Handbook (13 points). A knowledge score was calculated out of 20 points by adding the number of danger signs recalled and the number of correct responses to the 13 questions.

Additional variables that might influence women’s level of knowledge were collected. These included demographic characteristics (age, level of education of both partners, place of residence, type of family), clinical characteristics (gravidity, parity, gestational age, number of ANC visits, number of living children, past abortion, death of previous child under 5 years of age, previous stillbirth, previous surgery on reproductive tract and “bad obstetric history”), and whether pregnant woman had read the MCH Handbook.

### Sample size

In the absence of a baseline level of knowledge on obstetric danger signs among Bhutanese women, we calculated a sample size of 441 for a finite population of 24,846 based on the following assumptions: 50% probability for good knowledge, 5% margin of error, and 15% drop out rate.

### Data entry and analysis

Data were entered into EpiData Entry version 3.1 and analyzed using EpiData Analysis version 2.2.3 (EpiData Association, Odese, Denmark) and STATA version 13.1 (StataCorp LP USA). Numeric variables were reported as mean, standard deviations, median and interquartile range. Categorical variables were reported as frequencies and percentages. Normality for continuous variables was tested using Shapiro-Wilk test.

Knowledge was categorized as ‘good’ (≥80%, score 16–20), ‘satisfactory’ (60–79%, score 12-15) and ‘poor’ (< 60%, score ≤ 12). Given the high importance attached to increasing pregnant women’s level of knowledge on obstetric danger signs to reduce ‘first delay', we decided on a higher threshold to categorize knowledge as ‘good'.

Pearson’s correlation coefficient was calculated between knowledge score as well as number of danger signs recalled and woman’s age, period of gestation, gravidity and parity. Association between knowledge score and participant characteristics were tested using t-tests (and corresponding non-parametric, Kruskal-Wallis test) for numeric variables (mother’s age, gravidity, parity, number of living children, abortions and stillbirths in previous pregnancies, deaths of previous child under 5 years of age, gestational age, number of ANC visits, and number of past abortions).

Associations between level of knowledge ('good’ versus ‘satisfactory’ and ‘poor’ combined) and socio-demographic characteristics (age of mother, level of education of both partners, place of residence, family type, having read MCH Handbook) and clinical characteristics (death of child under 5 years of age, previous surgery on reproductive tract) were assessed with odds ratios (OR) using a log-binomial regression model. Those factors with a *p*-value < 0.1 during the unadjusted analysis were included in the final adjusted analysis. All results with *p* < 0.05 were considered significant.

### Ethics considerations

Ethics clearance was obtained from the Research Ethics Board of Health, Ministry of Health, Bhutan. Informed written consent was taken from each participant prior to the interview.

## Results

A total of 422 pregnant women were interviewed (response rate = 96.0%). The mean age of participants (±SD) was 27.9 (±4.8) years. Almost half of the women (196, 46.4%) were in their first pregnancy and the median gestational age was 34 weeks (IQR 26, 38). Three-quarters of the women (309, 73.2%) were in the third trimester of their pregnancy and a quarter of them (112, 26.5%) had come for their 7^th^ – 10^th^ ANC visits.

In their past obstetric history, one woman was admitted for gestational hypertension or pre-eclampsia, three women had experienced stillbirths and 13 women reported at least one child who had died under 5 years of age. Among multigravid women, none had a “bad obstetric history” (Rh incompatibility of parents’ blood groups, three or more consecutive spontaneous abortions, birth weight of past child less than 2500 g or more than 4500 g). Table 1 summarizes the basic demographic and clinical characteristics of the participants.

**Table 1:** Demographic and clinical characteristics of pregnant women attending antenatal clinic at Gyaltsuen Jetsun Pema Mother and Child Hospital, Thimphu, Bhutan, May 2019 – July 2020 (*n* = 422)

Basic characteristics	n (%)
**Total**	**422 (100)**
Mother’s age (years)	
18–24	107 (25.4)
25–34	276 (65.4)
35–40	39 (9.2)
Mother’s level of education	
None	47 (11.1)
Non-formal education	10 (2.4)
Primary	26 (6.2)
Secondary	243 (57.6)
Tertiary	96 (22.7)
Father’s level of education	
None	41 (9.7)
Primary	33 (7.8)
Secondary	201 (47.7)
Tertiary	147 (34.8)
Residence	
Urban	376 (89.1)
Rural	46 (10.9)
Type of family	
Extended	223 (52.8)
Nuclear	199 (47.2)
Current gravida	
Primigravid	196 (46.4)
Two to four	218 (51.7)
Five or more	8 (1.9)
Trimester during visit	
1^st^ trimester	6 (1.4)
2^nd^ trimester	107 (25.4)
3^rd^ trimester	309 (73.2)
Number of ANC visits at the time of interview	
1–3 visits	119 (28.2)
4–6 visits	191 (45.3)
7–10 visits	112 (26.5)
Number of past abortions^a^	
0	368 (87.2)
1	42 (10.0)
2	12 (2.8)
Surgery on reproductive tract in past^b^	
No	394 (93.4)
Yes	28 (6.6)

^a^Primigravida women were excluded

^b^Surgeries included caesarean section, salpingotomy, salpingectomy, myomectomy etc

*ANC* antenatal care

### Sources of information on danger signs

**Fig. 1:**
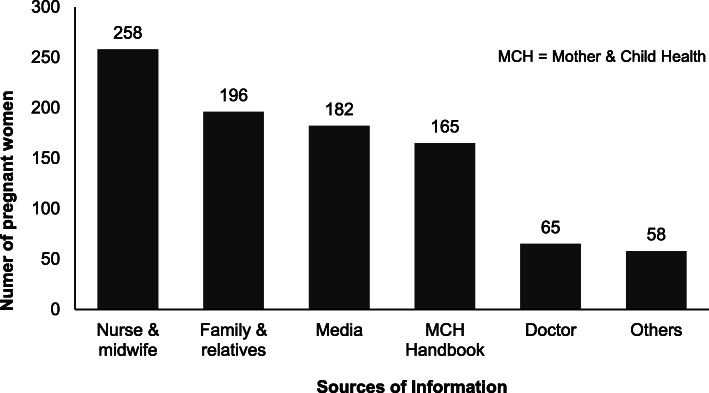
Sources of information on obstetric danger signs reported by pregnant women ‘who had heard about obstetric danger signs’ attending antenatal clinic at Gyaltsuen Jetsun Pema Mother and Child Hospital, Thimphu, Bhutan, May 2019 – July 2020 (*n* = 335)

Over three-quarters of women (335, 79.4%) had heard about obstetric danger signs. Most women cited ‘Nurse/midwife’ as their source of information on danger signs (258, 77.0%). Among 375 literate women, 308 (82.1%) reported having read the MCH Handbook and 165 (44.0%) reported the MCH Handbook as a source of information on obstetric danger signs. Figure 1 lists the frequency of each source cited by the pregnant women as their source(s) of information regarding obstetric danger signs.

### Knowledge of obstetric danger signs

The mean knowledge score (±SD) was 12.0 (±2.5) out of 20. Twenty women (4.7%) had ‘good’ knowledge on obstetric danger signs. Most women (245, 58.1%) had ‘satisfactory’ knowledge while a third of them (157, 37.2%) had ‘poor’ knowledge. The median number of danger signs recalled was 2 (IQR 1, 3). Among the 335 women who reported having heard of danger signs previously, 68 (20.3%) could not recall any danger sign. Most women recalled vaginal bleeding (227, 67.8%) while very few women recalled symptoms of pulmonary embolism (6, 1.8%) among the seven danger signs.

**Fig. 2:**
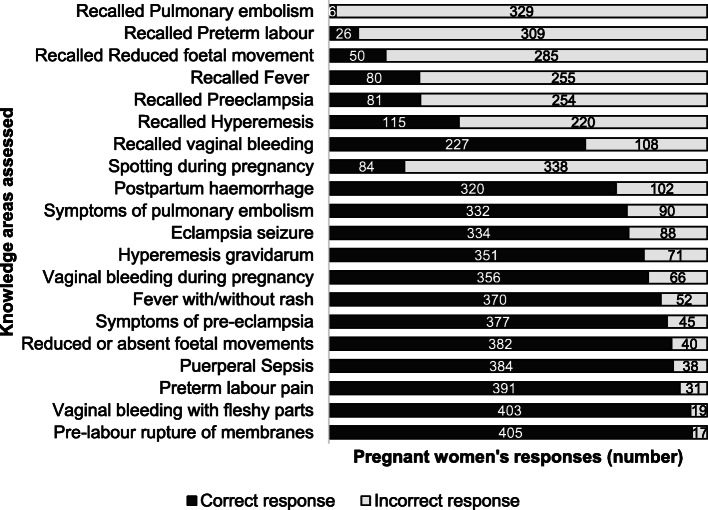
Responses to questions related to knowledge of obstetric dangers signs by pregnant women attending antenatal clinic at Gyaltsuen Jetsun Pema Mother and Child Hospital, Thimphu, Bhutan, May 2019 – July 2020 (*n* = 422)

Of the 13 questions, over 90% of women chose the correct response to five questions related to: pre-labour rupture of membranes (96.0%), vaginal bleeding with fleshy parts (95.5%), preterm labour pain (92.7%), puerperal sepsis (91.0%) and reduced foetal movements (90.5%). Three questions with most incorrect answers were related to: spotting during pregnancy (19.9%), postpartum haemorrhage (75.8%) and symptoms of pulmonary embolism (78.6%). The details of knowledge assessment using the 20 items are shown in Fig. 2.

### Factors associated with knowledge of obstetric danger signs

Knowledge score (r = 0.17, *p* < 0.001) and number of danger signs recalled (*r* = 0.12, *p* = 0.033) had significant correlation with the period of gestation. There was a significant association, based on Kruskal-Wallis test, between the number of danger signs recalled with mother’s level of education (*p* < 0.001), father’s level of education (*p* = 0.011) and having read the MCH handbook (*p* < 0.001). Level of knowledge had significant association, based on t-test, with number of ANC visits (*p* = 0.002) and gestational age (*p* = 0.033).

In the unadjusted analyses, having read the MCH Handbook (OR 8.9, 95% CI 1.1–73.8 *p* = 0.043), previous surgery on the reproductive tract (OR 3.9, 95% CI 1.2–12.7, *p* = 0.022) and father’s level of education (OR 15, 95% CI 1.4–163.2, *p* = 0.026) had significant association with ‘good’ level of knowledge. However, in the adjusted analysis, only one factor – ‘previous surgery on the reproductive tract’ – had significant association (OR 5.1, 95% CI 2.5–10.1, *p* < 0.001) with ‘good’ level of knowledge. Table 2 summarizes the results of the logistic regression analysis.

**Table 2:** Factors associated with knowledge of obstetric danger signs among pregnant women attending antenatal clinic at Gyaltsuen Jetsun Pema Mother and Child Hospital, Thimphu, Bhutan, May 2019 – July 2020 (*n* = 422)

Variable	Good Knowledge	Poor and Satisfactory Knowledge	Unadjusted analyses		Adjusted analyses	
	n (%)	n (%)	OR (95% CI)	***p*** value	aOR (95% CI)	***p*** value
Mother’s age group						
18 to 24 years	2 (2)	105 (98)	0.3 (0.1–1.4)	0.122	Ref	
25 to 34 years	16 (6)	260 (94)	Ref		9.2 (0.0–25,675)	0.584
35 to 40 years	2 (5)	37 (95)	0.9 (0.2–4.0)	0.866	9.6 (0.0–26,811)	0.576
Mother’s level of education						
None	0 (0)	47 (100)	Ref			
Non-formal education	2 (20)	8 (80)	1.2 (0.1–17.6)	0.869		
Primary education	0 (0)	26 (100)	–	–		
Secondary education	10 (4)	233 (96)	0.2 (0.0–2.0)	0.178		
Graduate education	7 (8)	83 (92)	0.4 (0.0–4.1)	0.458		
Others^a^	1 (17)	5 (83)	–	–		
Father’s level of education						
None	1 (2)	40 (98)	Ref		Ref	
Primary education	0 (0)	33 (100)	–	–	0.0 (0–0)	0.998
Secondary education	6 (3)	195 (97)	1.2 (0.1–10.5)	0.849	0.5 (0.0–15.4)	0.669
Graduate education	10 (7)	126 (93)	3.2 (0.4–25.6)	0.278	2.8 (0.2–44.9)	0.460
Others^a^	3 (27)	8 (73)	15 (1.4–163.2)	0.026	13.4 (0.7–239.4)	0.077
Place of Residence						
Rural	2 (4)	44 (96)	Ref			
Urban	18 (5)	358 (95)	1.1 (0.2–4.9)	0.895		
Death of child aged < 5 years	1 (8)	12 (92)	1.7 (0.2–13.8)	0.615		
Surgery on reproductive tract	4 (14)	24 (86)	3.9 (1.2–12.7)	0.022	**5.1 (2.5–10.1)**	**< 0.001**
Family type						
Nuclear	10 (5)	189 (95)	Ref			
Extended	10 (4)	213 (96)	0.9 (0.4–2.2)	0.794		
Read MCH Handbook						
No	1 (1)	103 (99)	Ref		Ref	
Yes, some of them	12 (5)	218 (95)	5.7 (0.7–44.2)	0.098	–	–
Yes, all of them	7 (8)	81 (92)	8.9 (1.1–73.8)	0.043	–	–

^a^Includes post-secondary certificates or diplomas and post-graduate degrees including masters or doctorates

*OR* Odds Ratio, *aOR* Adjusted Odds Ratio, *CI* Confidence Interval, *MCH* Mother and Child Health

## Discussion

### Knowledge of obstetric danger signs

Most women in our study had low scores on recall of obstetric danger signs. However, when presented with an obstetric emergency, most recognized the urgency of the situation and identified the need to seek medical attention. Most women were knowledgeable about pre-labour rupture of membranes, vaginal bleeding with fleshy parts and preterm labour. Most women had poor knowledge on spotting during pregnancy, postpartum haemorrhage and symptoms of pulmonary embolism.

Studies in the past have equated women’s knowledge on obstetric dangers signs with their ability to spontaneously recall varying number of danger signs [13–15, 19–22]. These studies surveyed women in the community [13, 14, 20, 22, 23] at different times after their last pregnancy [13–15] and assessed danger signs by classifying them as related to antepartum-, intrapartum- and postpartum-period [14, 19, 20, 23]. Though we cannot directly compare the levels of knowledge, the median number of danger signs recalled in our study is comparable to that reported in Tanzania [13]. The most common danger sign recalled in our study was vaginal bleeding, a pattern that is similar to what is reported in other studies [13, 15, 20–22, 24, 25]. However, the most common danger sign recalled was fever in Madagascar and Papua New Guinea while abdominal pain was the most common danger sign recalled in Bangladesh [26].

### The role of MCH handbook

In our study, having read the MCH Handbook had significant association with the number of danger signs recalled. However, it did not influence the overall knowledge score on obstetric danger signs. A 2018 recommendation by the World Health Organization (WHO) for home-based records for maternal, newborn and child health reported that home-based records empower women to make decisions by improving their knowledge [27]. WHO also reported increased healthcare-seeking patterns for antepartum and postpartum complications in places where some form of home-based records were used [27]. The MCH Handbook of Bhutan replaced three previously used home-based records: 1) antenatal record card, 2) postnatal clinic card, and 3) child immunization and growth chart card [17]. It also incorporates health education and information related to aspects of pregnancy, breastfeeding as well as child growth and development [10, 17, 18]. The present MCH Handbook used in Bhutan fulfils all three levels of functions expected of a home-based record: data recording and storage function, behaviour change communication function, and monitoring and referral function. However, the digital tracking of pregnancy through the MCH Handbook only captures how frequently pregnant women come for their ANC and postnatal visits but not the quality of each visit.

### Factors associated with levels of knowledge

In our study, having undergone surgery in the past (caesarean section, salpingectomy, salpingotomy, myomectomy) had five-times higher odds of having ‘good’ knowledge on obstetric danger signs, a finding similar to that reported in Uganda [23]. Both knowledge score and number of danger signs recalled had significant correlation with the period of gestation in our study. Studies elsewhere have reported increased knowledge with more ANC visits [19] and increased utilization of ANC services [22]. Health education increases awareness of danger signs [21, 28] and is reported to have doubled the odds of knowing about danger signs [14].

The level of education of both the mother and father influenced the number of danger signs recalled but did not impact their overall knowledge score. Other studies have reported increased odds of knowing and recalling danger signs with higher levels of mother’s education [19, 21, 28]. Similarly, the number of danger signs recalled by a pregnant woman was also associated with her husband’s level of education [12]. Educating husbands together with women might be a method to reinforce understanding of danger signs during pregnancy. A multi-centre study among pregnant women admitted in late pregnancy to hospitals in 2019 noted that women-invited husband participation was an enabler for early ANC booking and subsequent visits in Bhutanese women [10]. Age of women, place of residence and parity are other factors that have been reported by others to have a significant association with knowledge of danger signs [22, 28].

Knowledge of danger signs during pregnancy is one of the components of birth preparedness and complication readiness [29]. Those with knowledge of at least one danger sign were twice as likely to have better birth preparedness plans [20, 25, 26] and four-times more likely to be aware of birth preparedness and complication readiness [23]. In Nepal, women and their families failed to seek care in time during obstetric emergencies as they were unaware of danger signs [30].

### Reducing maternal mortality

Disseminating information through the media could improve knowledge of danger signs as only half of women in our study reported media as a source of information on danger signs. However, ‘good’ knowledge of danger signs alone without easy access to health facilities would hinder receiving timely services. Geographical inaccessibility has been reported as a barrier to using ANC services in Bhutan [10] with women in rural areas twice as likely to deliver at home than their urban counterparts [31].

Achieving the Sustainable Development Goals requires an integrated approach as health shares an intricate and reciprocal relationship with other socioeconomic and environmental factors [3]. Income inequality and poverty alleviation still remain barriers to timely and quality care: women in Bhutan intentionally delayed their ANC booking visit due to lack of funds [10]. As Bhutan graduates from a least developed country to a lower-middle income country, more investment in health could include pre-conception care and effective implementation of the child’s first 1000 golden days programme.

### Limitations

The high rate of correct responses to most questions could have been due to respondent’s social desirability bias where the respondents may have felt compelled to choose “seek care from a hospital” or simply “guessed” it to be the expected response.

## Conclusion

Most pregnant women had ‘satisfactory’ knowledge of obstetric danger signs. Although explicit recall of danger signs was poor, women recognized obstetric emergencies and identified the appropriate action warranted.

## Supplementary Information

**Additional file 1.**

## Data Availability

The dataset used for this study (minus key identifiers) are available from the corresponding author upon request.

## Abbreviations

ANC: Antenatal Care
MCH: Mother and Child Health
MMR: Maternal Mortality Ratio
