# Supplementary material for: Knowledge and understanding of obstetric danger signs among pregnant women attending the antenatal clinic at the National Referral Hospital in Thimphu, Bhutan: a cross-sectional study
Source: BMC Pregnancy Childbirth. 2021 Feb 2;21:104. doi: 10.1186/s12884-021-03580-4 (PMC7852084; doi:10.1186/s12884-021-03580-4)
Supplement: Supplementary file 1 — Additional file 1. [file 12884_2021_3580_MOESM1_ESM.pdf]

(Data extraction form)

Knowledge of Obstetric Danger Signs - JDWNRH

Information to be extracted from the MCH HANDBOOK or the MCH REGISTER + Interview

**Serial no**		
1	Date of interview	____________________ (dd/mm/yyyy)
2	Date of birth of mother	____________________ (dd/mm/yyyy)
3	Level of education of mother	☐ None<br>☐ Non-formal education<br>☐ Primary education<br>☐ Secondary education<br>☐ Graduate education<br>☐ Others
4	Level of education of father	☐ None<br>☐ Non-formal education<br>☐ Primary education<br>☐ Secondary education<br>☐ Graduate education<br>☐ Others
5	Residence	☐ Urban<br>☐ Rural
	**Past obstetric history**	
6	Mother is Rh negative and father is Rh positive	☐ No<br>☐ Yes
	History of three or more consecutive spontaneous abortions	☐ No<br>☐ Yes
	Baby weighing less than 2500 grams	☐ No<br>☐ Yes
	Baby weighing more than 4500 grams	☐ No<br>☐ Yes
	Last pregnancy: admission for hypertension, preeclampsia, eclampsia	☐ No<br>☐ Yes

	Previous surgery on reproductive tract (caesarean section, cervical cerclage, cone biopsy, myomectomy, ectopic pregnancy)	☐ No<br>☐ Yes
	**Description of current pregnancy**	
7	Gravida	
	Parity	
	Living children	
	Abortion in previous pregnancy	
	Stillbirth in previous pregnancy	
	Death of any previous child	
8	Number of ANC visits	
9	Period of gestation	_____ + __ (xx+x)
	**ADDITIONAL DETAILS (TO BE INTERVIWED HERE ONWARDS)**	
10	Type of family	☐ Extended<br>☐ Nuclear<br>☐ Single-family
11	Have you heard of danger signs during pregnancy?	☐ No<br>☐ Yes
12	If yes to above question, then where did you hear about them? *(You may select multiple responses)*	☐ Nurse/mid-wife<br>☐ Doctor<br>☐ Family and relatives<br>☐ Media (television, newspaper)

		☐ MCH Handbook<br>☐ Others
13	Have you read (or has someone read to you) the MCH Handbook on obstetric danger signs?	☐ No<br>☐ Yes, some of them<br>☐ Yes, all of them
	**KNOWLEDGE ON OBSTETRIC DANGER SIGNS**	
14	Can you list any of the seven danger signs listed in the Maternal and Child Health Handbook? *(Wait for the mother to answer)*	☐ *Per vaginal bleeding*<br>☐ *High fever*<br>☐ *Labour pains before 37 weeks*<br>☐ *Severe vomiting or severe abdominal pain*<br>☐ *Severe headache, blurred vision or convulsion*<br>☐ *Fast or difficult breathing*<br>☐ *Baby not moving or moving less than normal*
15	Spots of blood in your panties during your pregnancy is normal.	☐ Yes<br>☐ No<br>☐ Don’t know
16	Vaginal bleeding that is similar to menstrual flow is normal during pregnancy.	☐ Yes<br>☐ No<br>☐ Don’t know
17	Vaginal bleeding with passage of fleshy parts, what is the next step you should do?	☐ You can stay home for next few hours<br>☐ Come to hospital as soon as possible<br>☐ Don’t know
18	During your pregnancy, if you have fever with or without reddish dots or patches on your skin, what should you do?	☐ Take paracetamol<br>☐ Take rest and lie down<br>☐ Come to hospital<br>☐ Don’t know
19	Labour is the process of giving birth and is associated with pain and delivery of the baby. During labour, if your water breaks at home, what is the next step you should do?	☐ You can stay home for next few hours<br>☐ Come to hospital as soon as possible<br>☐ Don’t know
20	Labour is the process of giving birth and is associated with labour pain. If labour occurs before 37 weeks of gestation, what will you do?	☐ Will come to hospital<br>☐ I can manage the pain with pain killers<br>☐ Don’t know

21	During the time when you are pregnant, if you have severe headache, blurring of vision or some abnormal sensation in your tummy, what should you do?	☐ Take rest and lie down<br>☐ Immediately come to hospital<br>☐ Don't know
22	During the time when you are pregnant, if you seizures (involuntary movement of limbs/mouth, with or without loss of consciousness, followed by drowsiness/headache), what should you do?	☐ Lie down on your right side<br>☐ Immediately come to hospital<br>☐ Don't know
23	If you have repeated bouts of vomiting during your pregnancy so much so that it does not allow you to eat or have a proper meal, what should you do?	☐ Take anti-vomiting medicines from shops<br>☐ Seek care from a hospital<br>☐ Drink a lot of water and stay home<br>☐ Don't know
24	During pregnancy, it is normal to have some breathing difficulties. If you have a feeling of fast and unusually difficult breathing, what would you do?	☐ Take anti-vomiting medicines from shops<br>☐ Seek care from a hospital<br>☐ Drink a lot of water and stay home<br>☐ Don't know
25	For some period of times, the baby may sleep and may not move. Sometimes, if the baby does not move for long periods or if you feel that it moves less than normal, what would you do?	☐ Lie down on one side<br>☐ Come to hospital<br>☐ Don't know
26	After delivery and discharge from hospital, if you have vaginal bleeding that is foul smelling or that is persistent, what should you do?	☐ Maintain hygiene and stay home<br>☐ Wait until your regular postnatal visit<br>☐ Come to hospital<br>☐ Don't know
27	After delivery and discharge from hospital, if you have persistent abdominal pain and/or fever, what should you do?	☐ Take paracetamol<br>☐ Take rest and lie down<br>☐ Come to hospital<br>☐ Don't know

-End-
